# Early metabolic changes following ischemia onset in rats: An *in vivo* diffusion-weighted imaging and ^1^H-magnetic resonance spectroscopy study at 7.0 T

**DOI:** 10.3892/mmr.2015.3283

**Published:** 2015-01-30

**Authors:** GEN YAN, ZHUOZHI DAI, YINGHUA XUAN, RENHUA WU

**Affiliations:** 1 Department of Radiology, Affiliated Hospital, Jiangnan University, Wuxi, Jiangsu 214062, P.R. China; 2 Department of Radiology, The Second Affiliated Hospital, Shantou University Medical College, Shantou, Guangdong 515041, P.R. China; 3 Provincial Key Laboratory of Medical Molecular Imaging, Shantou, Guangdong 515041, P.R. China; 4 Department of Basic Medicine, Jiangnan University Medical School, Wuxi, Jiangsu 214122, P.R. China

**Keywords:** magnetic resonance spectroscopy, diffusion-weighted imaging, ischemic stroke, time window

## Abstract

Despite improvements in imaging techniques, it remains challenging to quantitatively assess the time of ischemic onset of an acute ischemic stroke. It is crucial to evaluate the early signs of infarction, which are predictive of responses to recombinant tissue plasminogen activator within a treatment window of 4.5 h after stroke induction. The aim of the present study was to assess and quantify the onset time for hyperacute middle cerebral artery occlusion (MCAO) ischemic stroke by measuring the apparent diffusion coefficient (ADC) of diffusion-weighted imaging (DWI) and ^1^H-magnetic resonance spectroscopy (MRS) at 7.0 T. DWI, conventional T_2_-weighted imaging (T_2_WI) and subsequent focal ADCs were employed to evaluate ischemic brain lesions in a rat model of MCAO (n=20) at different time-points following a stroke. A quantitation of local changes in metabolite concentrations within the lesions was performed using MRS. Proton metabolites were quantified automatically using LCModel software. At 30 min after MCAO, intense signals were observed in the DWI spectra of all animals. No abnormal signal was observed within 3 h by T_2_WI. ADC images of the central area, peripheral striping and on the fringes of the infarction demonstrated a lower signal than that of the normal side. The ADC decreased significantly within 30 min after infarction, followed by a gradual elevation in volatility levels and then becoming relatively stable at a lower level 3 h later. MRS exhibited a consistent elevation of lactate and reduced *N*-acetyl aspartic acid. Glutamate and taurine reached a maximum 2 h after MCAO and began to decrease 1 h later. In conclusion, the present study demonstrated that hyperacute ischemic stroke can be quantitatively detected with the application of ADC, DWI and MRS. These methods may also be used to quantitatively assess the ischemic onset time of a hyperacute stroke.

## Introduction

As the development of therapeutics for ischemic injury continues, brain imaging is taking an increasingly important role in the initial evaluation of patients with acute stroke (1). Exclusion of cerebral hemorrhage by computed tomography (CT) or magnetic resonance imaging (MRI) is necessary for any acute intervention in ischemic stroke (1,2). At present, the American Heart Association Stroke Council recommend the administration of intravenous recombinant tissue plasminogen activator (rtPA) within a 4.5-h thrombolytic therapy time window for patients with acute ischemic stroke (2,3). Despite its potential effectiveness, no objective assessment or quantitative criteria exist to evaluate factors governing the 4.5-h time window. Intracranial hemorrhage remains the major risk of intravenous rtPA treatment (2).

With the potential of rtPA treatment, interest in using CT to identify the preliminary changes of ischemic brain injury (early infarct signs) or arterial occlusion (hyperdense vessel signs), which may affect treatment decisions is increasing. In addition, sulcal effacement and loss of gray-white differentiation in the cortical ribbon (particularly at the lateral margins of the insula) or lentiform nucleus can often be detected within 6 h in up to 82% of patients with large-vessel anterior circulation occlusions (4). Such symptoms often result in poorer outcomes (5). Standard MRI sequences (T_1_ weighted, T_2_ weighted and proton density) often do not reveal changes resulting from acute ischemia (6,7). The mismatch between magnetic resonance (MR), diffusion-weighted imaging (DWI) and perfusion-weighted imaging (PWI) in the visualization of the penumbra has been suggested to guide thrombolytic therapy. However, several studies have indicated that, at least under certain circumstances, the initial diffusion abnormality is reversible and that visually thresholded perfusion volumes overestimate the penumbra (8,9). Sequential MRI studies performed on patients treated with thrombolytic therapy have revealed that an MRI may visualize the salvaged mismatch-defined penumbral tissue in smaller volumes of infarction among patients with successful recanalization (9).

It is well known that diffusion MRI and proton MR spectroscopy (MRS) can evaluate early metabolite alterations in acute stroke in humans and animals (10). Several studies have demonstrated that MRI is the modality of choice for studies of early stroke brain injury in humans (11) and in a rat middle cerebral artery occlusion (MCAO) model (12). MR-based DWI is sensitive to changes in the magnitude and direction of water diffusion. Numerous studies have demonstrated that the apparent diffusion coefficient (ADC), a parameter derived from DWI, is reduced in the hyperacute phase of an ischemic event, resulting from the influx of water from the extracellular space into the intracellular space (13,14). Proton MRS can provide information on the severity of ischemic injury by measuring the levels of metabolites following cerebral ischemia (15). In particular, a decrease in *N*-acetyl-aspartate (NAA) has been observed to correlate with neuronal loss and is a predictor of outcome following stroke and traumatic brain injury (16). Recent studies on ischemic mice or human patients with traumatic brain injury revealed that important metabolite concentration changes occurred prior to the appearance of abnormalities and which could not be detected by MRI (17), suggesting that MRS is better at detecting early tissue damage or compromised cell function. Following the progression of ischemic injury, proton MRS may be a valuable tool for providing information on the timing and pattern of acute metabolic changes, as increased quantities of lactate are detected in the early stages of stroke and are prognostic (18).

In the present study, a rat model of MCAO was used to continuously assess and quantify brain alterations at 1-h intervals within the first 3 h after stroke onset using multiparametric MRI. The early neurochemical changes in ischemic brain tissue and their role in early ischemic damage *in vivo* were further investigated.

## Materials and methods

### Animal model

Adult male Sprague-Dawley rats, aged 7–8 weeks old and weighing 250–280 g, were used in all experiments. Rats were obtained from the Shantou University Medical College Laboratory Animal Center (Shantou, China). The animals were housed under standard laboratory conditions with a 12-h light/dark cycle at a room temperature of 24±1°C and a relative humidity of 45±5%. All experimental procedures were approved by the Care of Experimental Animals Committee of Shantou University (Shantou, China) and were performed in accordance with their guidelines. Permanent middle cerebral artery occlusion (pMCAO) was induced using a previously described method of intraluminal vascular occlusion (19). Rats subjected to pMCAO were subjected to a consistent and reproducible ischemic lesion in the unilateral striatum and cortex. Briefly, a 20-mm incision was made at the center of the neck and the right common carotid artery, external carotid artery (ECA) and internal carotid artery (ICA) were exposed under an operating microscope (SXP-1C; Shanghai Medical Equipment Co., Shanghai, China). The ECA and ICA were temporarily clamped using microsurgical clips. A 5-0 silk suture was tied loosely at the origin of the ECA and ligated at the distal end of the ECA. A length of 4-0 monofilament nylon suture (18.5–19.5 mm), determined by the animal’s weight and with its tip rounded by heating near a flame, was introduced into the ECA lumen through a small puncture. The silk suture surrounding the ECA origin was tightened around the intraluminal nylon suture to prevent bleeding and the microsurgical clips were removed. The nylon suture was gently advanced from the ECA into the lumen of the ICA until it blocked the origin of the MCA. Immediately following the occlusion, the rat was positioned in an animal holder equipped with a birdcage radio frequency coil manufactured in house.

### MRI and localized proton MR spectroscopy acquisition

For *in vivo* MRI and ^1^H MRS, animals were initially anesthetized using a chamber pervaded with 5% isoflurane (Sigma-Aldrich, St. Louis, MO, USA) in oxygen. Subsequently, the mice were anesthetized through a mask by spontaneous inhalation of 1.5–2.0% isoflurane and air using an anesthesia unit. Anesthetized rats were placed in the prone position with the head firmly fixed on a palate holder equipped with an adjustable nose cone. MR imaging was performed on 12 animals using a 7.0 T horizontal DriveDrive 2 MR system (Agilent Technologies, Santa Clara, CA, USA) with a 160-mm bore magnet and a 400 mT/m actively shielded gradient coil. For proton spectra signal excitation and reception, a dedicated animal brain surface coil (20 mm, 157–350 MHz, Agilent Technologies) was used.

A scout view was initially obtained to verify the animal’s position and the image quality. T_2_ weighted images (sequence, rapid acquisition with a relaxation enhancement; repetition time (TR)/echo time (TE), 2,000/48 ms; number of acquisitions, 4; slice thickness, 1.0 mm; matrix, 192×192) were obtained. Diffusion-weighted indices were acquired from a multi-shot fast spin-echo trace sequence (TR, 3,000 ms; TE, 36 ms; b, 1,000 s/mm^2^; Δ,14.65 ms; duration, 5 ms, average, 4; matrix, 128×128). *In vivo* proton spectra volumes of interest (VOI) were positioned in regions with ischemic lesions (3.5×3.5×3.5 mm) based on multi-slice axial diffusion-weighted images. The VOI was adjusted to minimize intracranial lipid contamination. Localized shimming in the VOI was performed automatically based on three dimensional field mapping, leading to a water line width ranging from 15 to 20 Hz. An ultra short echo time stimulated echo acquisition pulse sequence was used for acquisition of the proton spectra in the VOI (TR/mixing time/TE, 5,000/12.72/2.35 ms; spectral width, 5,000 Hz; number of excitations, 160; scan time, 13 min). The water signal from the VOI was suppressed by variable power radio frequency pulses with optimized relaxation delays. To compensate for eddy currents, a water reference scan was acquired, which also served as an internal reference for absolute quantification.

*In vivo* proton spectra were analyzed using the LCModel 6.3-1B software (LCModel Inc., Oakville, ON, Canada) (20), which calculates the best fit to the experimental spectrum as a linear combination of model spectra (simulated spectra of brain metabolites). Raw data free induction decays were used as the standard data input. The water-suppressed time domain data were analyzed between 0.2 and 4.0 ppm without further T_1_ or T_2_ correction. The following 17 metabolites were included in the basis set: Alanine (Ala), aspartate (Asp), creatinine (Cr), γ-aminobutyric acid (GABA), glucose (Glc), glutamate (Glu), glutamine (Gln), glutathione (GSH), glycerophosphorylcholine (GPC), phosphorylcholine (PCho), myo-ionositol (mIns), lactate (Lac), *N*-acetyl aspartate (NAA), *N*-acetylaspartylglutamate (NAAG), phosphocreatine (PCr), scyllo-inositol (Scy) and taurine (Tau). In addition, macromolecules were also included in the basis set. To obtain more reliable results, only the sums of metabolites (NAA+NAAG, Glu+Gln, GPC+PCho and Cr+PCr) were estimated. The other metabolites, including mIns and Tau, were individually quantified.

The standard error estimates and Cramer Rao lower bounds (CRLBs) were used to provide useful estimates of reliability and uncertainty for each metabolite peak in the LCModel spectra (20). CRLBs have been used to provide acceptable reliability for estimates of fitting uncertainty.

### Statistical analysis

The SPSS software package version 12.01 (SPSS, Inc., Chicago, IL, USA) was used for statistical analysis. Data for each metabolite were assessed for homogeneity of variance and a one-way analysis of variance with a Bonferroni *post hoc* test was used to compare the means of the metabolite ratios for each time-point group. P<0.05 was considered to indicate a statistically significant difference.

## Results

**Figure 1:**
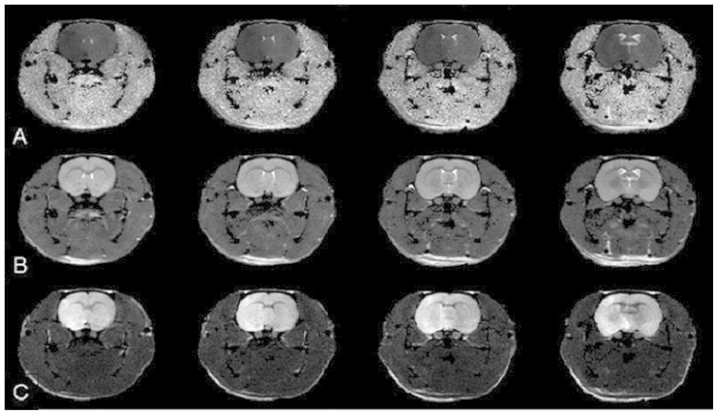
Slices of (A) T_2_-weighted image, (B) DWI and (C) ADC map of a rat 10 min after middle cerebral artery occlusion. Hyperintense lesions in the right corpus striatum and parietal cortex are clearly identified on DWI, and a corresponding reduction in ADC values (hypointense) was observed on the ADC map, whereas no lesion signs were observed on the T_2_-weighted image acquired 10 min after ischemia onset. ADC, apparent diffusion coefficient; DWI, diffusion-weighted imaging.

**Figure 2:**
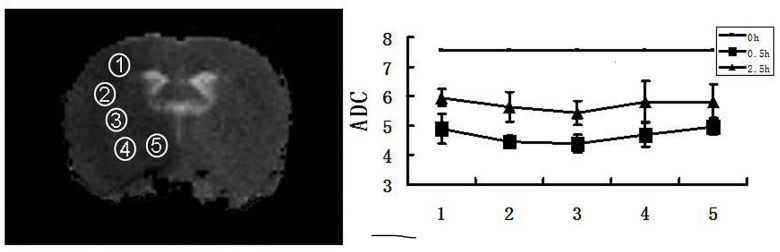
Alterations in the ADC values in damaged areas within 3 h of ischemia onset. The ADC significantly decreased in all damaged areas at 0.5 and 2.5 h after ischemia onset. At each time-point, the ischemic area exhibited gradually reduced ADC values from the periphery to the center (3, center). ADC, apparent diffusion coefficient.

**Figure 3:**
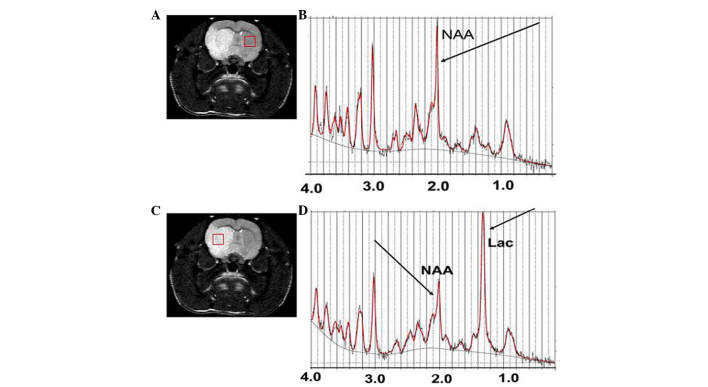
^1^H-MRS of a normal rat and ^1^H-(A) *In vivo* ^1^H-NMR spectra acquired from the corresponding brain regions. (B) Multislice diffusion-weighted axial images of the brain of a rat with MCAO with the volumes of interest centered in the ischemic lesion regions. Representative spectra were obtained from the ischemic lesion regions of each group (3.5×3.5×3.5 mm) and were fitted using the LCModel. Spectra were acquired with an ultra-short echo-stimulated echo acquisition pulse sequence (echo time/repetition time/average=2/5,000/160). (C) ^1^H-MRS of the brain of a normal rat and (D) ^1^H-MRS of the brain of a rat with MCAO 1 h after ischemia onset. The Lac peak (1.33 ppm) was significantly higher and its area was larger in the MRS of the rat with MCAO, while the NAA peak (2.02 ppm) was lower and its area was smaller as compared to that in the MRS of the normal rat. MCAO, middle cerebral artery occlusion; MRS, magnetic resonance spectroscopy; NMR, nuclear magnetic resonance; Lac, lactate; NAA, *N*-acetyl aspartate.

**Figure 4:**
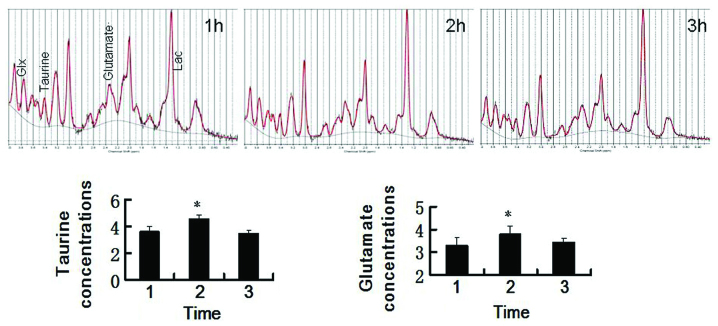
^1^H-MRS of a rat with MCAO 1, 2 and 3 h after the onset of ischemia. Taurine and glutamate concentrations in the ischemic regions at different time-points following MCAO are shown. Error bars indicate the standard deviation (n=10/group). ^*^P<0.05 vs. 1 and 3 h post ischemia. MCAO, middle cerebral artery occlusion; MRS, magnetic resonance spectroscopy.

For all 20 MCAO rats, the infarct was not observable by T_2_WI within 6 h from the onset of ischemia. However, abnormal hyperintensity signals were observed by DWI in the striatum and parietal cortex of all MCAO rats 10 min and 6 h after the onset of ischemia. The ADC demonstrated abnormal, hypointense signals in the same area and at the same time-point (Fig. 1). The baseline mean ADC was (7.573±0.553)×10^−4^ mm^2^/sec, comparable to values previously reported (21). At 30 min after MCAO, the ADC decreased from (4.7571±1.423)×10^−4^ to (5.7318±1.031)×10^−4^ mm^2^/sec. The ADC values increased gradually from the center towards the periphery of the damaged areas at the 0.5 h and 2.5 h time-points. At 3 h after the onset of ischemia, the ADC values were the same across all areas (Fig. 2). Typical water-suppressed proton MR spectra of the rat brain from the region of the ischemic lesion and the ipsilateral hemisphere (Fig. 3) represent the spectral quality consistently achieved in the present study. Generally, short echo time localization methods minimize T_2_ relaxation effects, which increases the sensitivity and reliability of the metabolite quantification (22). The high spectral resolution allowed for unambiguous signal assignment. In addition to the commonly observed ^1^H-MRS signals of the methyl resonances of NAA, Cr and Cho, spectral patterns of other metabolites, including Glu, Gln, GABA, mIns and Tau were discernible in the ^1^H-MRS spectra with a CRLB ≤15% from the two brain regions. The Lac peak was higher and the NAA peak lower in the central damaged area than that on the contralateral side at the 1 h time-point for all 12 MCAO rats (Fig. 3). ^1^H-MRS for each time-point after the onset of ischemia are shown in Fig. 4. Within the first 2 h, the concentrations of Glu and Tau increased gradually and then decreased significantly in the following 3 h (Fig. 4).

## Discussion

High DWI signal intensities in the right corpus striatum and parietal cortex of the Sprague-Dawley rats were observed following 30 min of middle cerebral artery occlusion in the present study, but no intensity changes were observed in the same regions by T_2_WI, even after 6 h of occlusion. The present finding was consistent with that of a previous study (23). DWI is a highly sensitive and specific technique for the early diagnosis of acute cerebral ischemia and is currently in use for the diagnosis and decisions on therapy of patients with stroke. Signal intensity in DWIs exhibited little change during the first hour after symptom onset and decreased thereafter, but all lesions remained hyperintense throughout the follow-up period. This pattern of signal intensity on DWI was most likely the result of the effects of two factors: Water diffusivity and intrinsic T_2_ properties (T_2_ shine-through) of the tissue being examined (24). Since the DWI signal remained high for a long period (up to three days in the present study), it is not ideal for estimating lesion age. However, the combined interpretation of T_2_WI and DWI may be insightful, as in cases where visual inspection of T_2_WI shows normal results while DWI shows highly abnormal signals, the stroke lesion age can be estimated as 6 h or less.

ADC is a parameter used for the quantitative assessment of the extent and severity of cerebral ischemic injury (13). A severe ADC decline in energy-depleted tissue can be explained by anoxic depolarization during MCAO, leading to an intracellular Na^+^ accumulation and water shift from the extracellular to the intracellular space (cytotoxic edema). Quantitative ADC voxel compartmental analysis revealed heterogeneity in the spatial-temporal profile of the ischemic penumbra and infarction. The ADC increased gradually from the center to the periphery of the damaged area at the 0.5 h and 2.5 h time-points after permanent MCAO. However, 3 h after the onset of ischemia, the ADC values were equivalent across the entire region. The present results indicated that an ADC map may provide useful information regarding the age of ischemic lesions at 3 h. These results provided a novel method to evaluate lesion age in a rat model of MCAO model, while previous methods were not sufficient to adequately and accurately characterize these conditions (14). Predictive algorithms also have the potential to serve as promising metrics for screening thrombolytics for acute ischemic stroke (4).

Glu is an excitatory neurotransmitter, which stimulates postsynaptic neurons, whereas GABA and Tau are inhibitory neurotransmitters that reduce the excitability of neurons. The homeostasis of the metabolism of Glu and GABA is usually dependent on the interaction between neurons and astrocytes under physiological conditions. Previous studies revealed that the concentrations of Glu, GABA and Tau decreased concurrently 4 and 8 h after MCAO, respectively (25,26). In the present study, the extracellular content of excitatory amino acids (Glu and Asp) and inhibitory amino acids (GABA and Tau) reached a maximum in rats 1–2 h after MCAO and began to decrease 3 h after MCAO, which was consistent with a previous study (27). Increases in extracellular Glu may lead to cell death by excitotoxicity, and therefore, it may be vital to detect Glu in patients with stroke in order to make prompt treatment decisions. The methods developed in the present study provide tools for screening patients for thrombolytic therapy. Combined analysis of DWI infarct size and location may provide important information about when Glu and Tau are at their highest levels, which may be used for selecting treatments for acute MCAO ischemic stroke within 2 h of onset.

**Figure 5:**
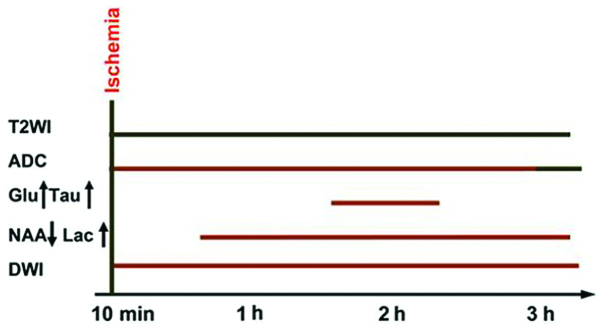
Magnetic resonance-based method to predict the onset time of ischemic lesions in animal models. Green (short-duration ischemia without a lesion) and orange bars (short-duration ischemia with a lesion) represent the period during which an abnormality was detected by the corresponding technique. DWI, diffusion-weighted imaging; NAA, *N*-acetyl aspartate; Lac, lactate; Glu, glutamate; Tau, taurine; ADC, apparent diffusion coefficient; T_2_WI, T_2_-weighted imaging.

These MR-based findings provide a relatively accurate method to predict the age of acute ischemic lesions in animal models (Fig. 5). Indeed, the final goal of our ongoing studies is to assess whether combined T_2_WI, ADC and MRS can be used for early diagnosis and to determine the onset time of acute ischemic stroke in human patients. Further studies are required to extend the present findings to humans. Precise knowledge of the onset time of thrombolytic stroke is important, but there are difficult questions based on the definition of acute ischemic stroke. The MR-based methods described in the present study are more objective than relying solely on the traditional image reading methods used by radiologists to estimate acute ischemic stroke lesion age and to select patients for treatment trials.

In conclusion, hyperacute cerebral infarction can be sensitively and specifically detected with the application of ADC on DWI and MRS. MR-based methods may also be used to quantitatively assess the ischemic onset time of hyperacute ischemic stroke. Objective and quantitative assessments of lesion age in hyperacute ischemic stroke are useful parameters for the identification of potential therapies in clinical trials and individualized treatments for stroke patients.
